# Growth of Femur after Resection of Knee-Joint

**Published:** 1891-01-10

**Authors:** 


### GROWTH OF FEMUR AFTER RESECTION OF KNEE-JOINT.

Dr. Ferd. Petersen exhibited at a meeting of the Medical Society of Berlin, a preparation showing the growth of the femur in a girl on whom resection of the knee-joint had been performed when she was eleven years old, and who died at the age of seventeen. The length of the bone when its growth from the lower epiphysis was thus arrested, was 8·5 centimetres, but the upper epiphysis thenceforth grew with abnormal rapidity, in fact a vicarious elongation took place from above. The canal of the nutritive artery in the sound limb ran from below, upwards, but in the other, after the cessation of growth in the lower end of the shaft, it was developed from above, downwards. The entire length of the resected limb was 1·7 cm. greater than that of the sound one. A consideration of this result is of great value in determining on the expediency of resection in childhood, while the growth of the skeleton is still in progress, since the chief objection to the operation, the shortening of the limb, is removed.

